# Causes of the male-female ratio of depression based on the psychosocial factors

**DOI:** 10.3389/fpsyg.2022.1052702

**Published:** 2022-11-16

**Authors:** Jun Tang, Tianyi Zhang

**Affiliations:** School of Foreign Studies, Hefei University of Technology, Hefei, China

**Keywords:** depression, male-female, Biopsychosocial Model, enTenTen20, causes

## Abstract

Recently, depression has been a great concern of researchers, and the research on the male-female ratio of depression mainly covers the fields of epidemiology and clinical studies. This study is made to explore the causes of the male-female ratio of depression from a humanistic perspective, namely, the psychosocial factors, thus providing some support and reference for further research on depression. Thereby, the authors select and analyze word lists closely collocated with “depression” according to word score and frequency through the word sketch in enTenTen20 by the corpus tool “Sketch Engine.” Meanwhile, the above words are further explored for the causes of the male-female ratio in depression from the biological, psychological, and social aspects based on the Biopsychosocial Model as well as some existing relevant medical research findings. Consequently, women are found to be more likely to suffer from emotional problems and depression than men under the influence of biological basis, general psychological characteristics, and social environment interference.

## Introduction

As a common social mental problem, depression has increasingly been an emotional obstacle to people and a frequently occurring disease in society. Meanwhile, depression is also one of the five major causes of disability and disease burden worldwide (Caspi et al., 2003).

Depression refers to a wide range of emotional problems, from mild negative emotions to serious emotional disorders, manifested by negative emotions such as sadness and distress, accompanied by behavioral characteristics such as withdrawal and inattention. Patients with severe depression also suffer from somatic symptoms such as insomnia and anorexia (Compas et al., 1993; Cassano and Fava, 2002). Relevant studies of depression have already brought about some enlightenment on the related prevention, mitigation, and treatment. As the gender differences in depression are less discussed with fruitful findings (Cao et al., 2013), the research on the causes of the male-female ratio in depression would be of great interest to researchers.

The gender difference in depression would occur as early as adolescence period. Women are more prone to depression; if this happens, the symptom of depression will develop until adulthood; and the male-female ratio in depression is about 1:2 (Solomon and Herman, 2009). Currently, most studies are mainly about clinical cases. In recent years, researchers have made studies on depression with neuroimaging (Huang et al., 2012; Mei and Qiu, 2018) and magnetic resonance brain functional imaging (Mei et al., 2019) to explore and analyze the gender differences in depression scientifically and empirically due to the development of medical science and technology. However, the exact etiology and pathological mechanism of depression are still under further exploration, and some previous researchers have assumed some biological hypotheses about depression (Shen and Liang, 2007). So far, most of the research on gender differences in depression has been conducted on epidemiology and clinical cases, such as incidence rate, suicide rate, and clinical symptoms (Khan et al., 2002; Blanco et al., 2012). Depression is both a medical problem for doctors to solve and a social problem for the general public to face; as a common psychological disease, depression involves many psychological and social factors, while few pieces of research are made on such factors.

Given the existing research progress and findings, analysis of depression from the field of the humanities may serve as a different impetus for possible further research to probe into the underlying psychological and social factors. Namely, some attempts could be made to explore complementary and supportive gender-related causes of depression based on the linguistic corpus data, which is an effective analysis tool of humanities and social sciences. Since the study on depression from the perspective of the humanities is in need and linguistic corpus data could provide some assistance for the prevention and treatment of depression, the two research questions are put forward as follows: (1) According to the retrieved corpus data of enTenTen20, are there any linguistic indicators related to the male and female sufferers of depression, respectively? If yes, which gender suffers more from depression? (2) Based on the corpus data and the Biopsychosocial Model, what causes the male-female ratio of depression?

## Materials and methods

As it is known to all, language, a system of communication that consists of a set of sounds and written symbols and is used by people of a certain community, not only reflects culture and society but also mirrors the world in which people live. And corpus, a large collection of written or spoken texts that is used for language research, could serve as an effective and supportive tool for the observation and analysis of human behavior, psychology, and so on, from the angle of linguistics.

To come up with answers to the above research questions, first, the Word Sketch tool in Sketch Engine is to be used for searching data related to “depression” from the linguistic corpus entitled enTenTen20; then, word relationships between “depression” and others are to be inferred from the means of corpus analysis from the linguistic perspective, namely, linguistic indicators of corpus data such as lexical frequency and collocation score; finally, selected linguistic indicators are to be analyzed based on the Biopsychosocial Model.

### EnTenTen20 corpus

EnTenTen20 corpus, one of the sub-corpora of the Tenten corpus family, is a new Internet corpus in the twenty-first century. As the latest and largest version currently, this corpus boasts a storage capacity of 38 billion words, covering online English downloaded texts from 2019 to 2021. Among the downloaded texts, the sample texts are from the largest network domain. Accounting for 40% of all corpus texts, the sample texts have been manually checked with all the poor-quality texts deleted. With timeliness and reliability, enTenTen20 is adopted for the sake of the validity of the analysis. What should be mentioned is that the data from the medical corpus failed to be adopted in this study, as there is no corresponding or related data for the discussion of the research questions in this study. Thus, the enTenTen20 is finally selected as the research data.

### Procedures

First, the word “depression” is selected as the search term. According to the Oxford Dictionary, “depression” means “a medical condition where a person feels very sad and anxious and often has physical symptoms such as being unable to sleep, etc.”; the second most commonly used meaning is “the state of feeling very sad and without hope,” referring to depressed mood and state; the third meaning is about the economic recession that is not related to the research questions and will be screened out in the data collection. In a word, the first two meanings meet the requirements of this research.

Second, the Word Sketch tool in Sketch Engine is used to search the term “depression” in enTenTen20. Before generating a word sketch, two types of parameters are defined—part of speech and lemma. The specific steps are conducted as follows: input “depression” in the input box for the basic morphology, select “noun” in the part of the speech box, and then click “show Word Sketch” to display depression matching results. In the end, there are 24 lists of different collocations being displayed, arranged from top to bottom according to the collocation score. In this way, the word sketch of “depression” based on the enTenTen20 corpus is conducive to the collocation behavior mode of “depression.”

Finally, in terms of contextual meaning related to causes of the gender difference of depression, the main pronouns and nouns of “depression,” the modifiers of “depression,” and the nouns modified by “depression” are sorted out and analyzed in terms of collocation score and lexical frequency.

### Lexical frequency and collocation score

Lexical frequency and collocation score, as explicit indicators of corpus data, are the fundamental means of corpus analysis from a linguistic perspective. Lexical frequency refers to the number of words in the text. The more this word is used, the higher frequency it is. High frequency means that the word is frequently used with the searched word, indicating a closer semantic relationship between them. Meanwhile, lexical collocation could indicate the syntactic-semantic co-occurrence of words. The “collocation” proposed by Firth is concerned with the theory that the meaning of a word is reflected in the words that accompany and co-exist with it (Firth, 1951). Linguists use “co-occurrence” to refer to a horizontal relationship. The higher the lexical co-occurrence, the closer the semantic relevance. Therefore, the closer semantic relationship between “depression” and some other words could be favorable for the inference of possible semantic and pragmatic logic. And the analysis of words’ meanings and different contexts would help with uncovering how and why the words are closely related. In short, through lexical frequency and collocation score, the relationship between depression and gender could be further analyzed in combination with medical theory.

### Biopsychosocial model

Engel. GL, a professor of Psychiatry and Internal Medicine at the University of Rochester School of Medicine, proposed a new medical model: Biopsychosocial Model in Science in 1977 (Engel, 1977). According to the connotation of the medical model proposed in the textbook Social Medicine of the Ministry of Education’s 10th 5-year Plan, the “Biopsychosocial Model” is regarded as the scientific concept and methodology of observing, analyzing, and thinking from biological, psychological, and social aspects when dealing with diseases and health problems, which means that psychological factors such as tension, anxiety, and depression, as well as social factors such as employment environment and economic income, health services and environmental pollution, also matter, in addition to biological factors such as bacteria, viruses, and parasites (Liang et al., 2004). Thereby, researchers could explore the etiopathogenesis from biological factors as well as individual psychological characteristics, psychological behavior, relevant socio-economic conditions, and other psychosocial factors that need to be taken into account. In other words, our body would imply biological, psychological, and social aspects simultaneously whether in a state of health or disease.

In a nutshell, the linguistic indicators of “depression” from the enTenTen20 corpus would be analyzed to explore the causes of depression effectively from the above-mentioned three types of factors of the medical model.

## Results

**FIGURE 1:**
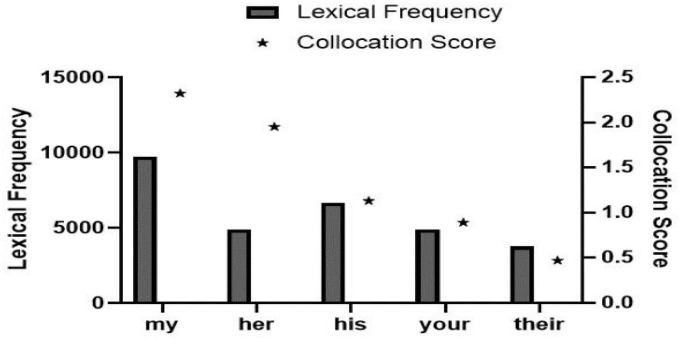
Pronominal possessors of depression.

**FIGURE 2:**
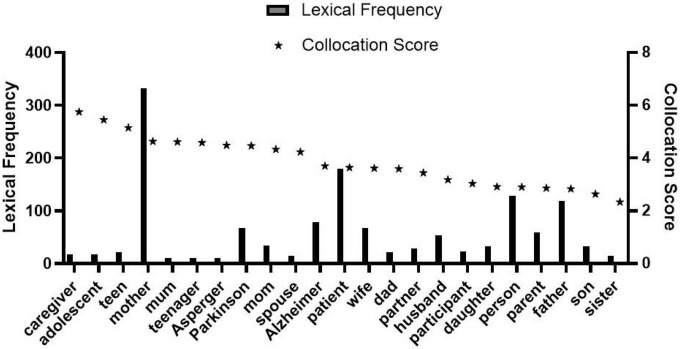
Possessors of depression.

**FIGURE 3:**
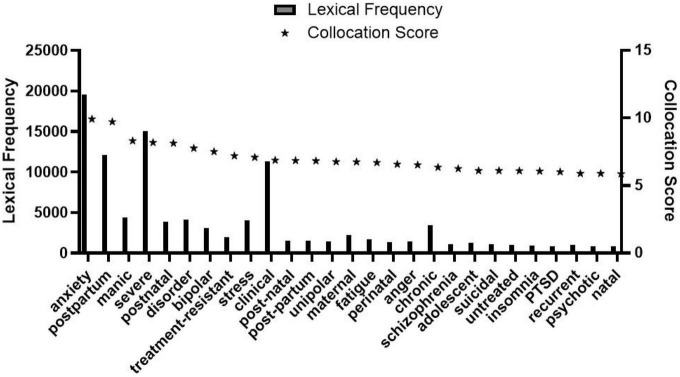
Modifiers of depression.

**FIGURE 4:**
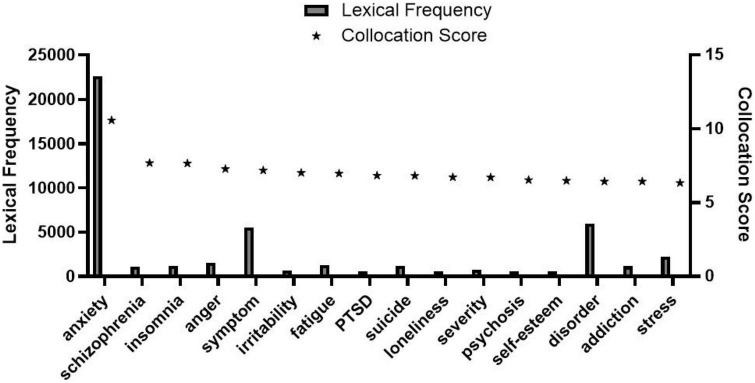
Nouns modified by depression.

In the word sketch of “depression” from the enTenTen20 corpus, the total number of retrieved “depression” reaches 1,163,138. And this reveals that the number of “depression” used as a noun is 1,163,138 in 18 wordlists with different collocations. In terms of the correlation between the word meanings and gender, we finally screened out four lists, including the list of pronominal possessors of depression, possessors of depression, modifiers of depression, and nouns modified by depression. Data at the top of the list is selected, and words unrelated (words related to the meaning of “economic recession”) are removed, to obtain explicit data related to the study. To show the data in an intuitively way, four Figures are finally drawn as follows:

As observed in Figure 1, collocation scores of “her” and “his” reflecting gender differences are 1.95 and 1.13, respectively, namely, the score of “her” is significantly higher than that of “his.” There are 23 lexical data in Figure 2. The frequency of the word “mother” is 332, which is very prominent compared with other words in the list. Through further comparison, we found that the collocation score and frequency of female-related words such as “mother,” “mum,” and “wife” are significantly higher than that of male-related words such as “Dad,” “father,” and “husband.” Totally 27 lexical data were collected in Figure 3, where “postpartum” has a frequency of 12,118 and a collocation score of 9.72, ranking relatively high. The frequency of other words related to female biological characteristics (such as “postnatal”) is also high. In addition, the data of the top 16 words modified by “depression” could be seen in Figure 4. Comparing Figure 3 with Figure 4, there are as many as six overlapping words, including “anxiety,” “stress,” “disorder,” “fatigue,” “anger,” and “insomnia,” which will be discussed in the following analysis.

## Discussion

The above statistical results referred indicate clearly that there are collocated words of gender-related depression sufferers, and more women are found to suffer from depression than men (observed from Figure 1); while those words related to the maternal role played by the female sufferers of depression also confirm such imbalance of the male-female ratio (observed from Figure 2). A conclusion could be made that the number of women suffering from depression is more than that of men, and there are differences between male and female sufferers of depression.

To explore the causes of such gender differences in depression, we made further analysis of the linguistic indicators of the lexical frequency and collocation score of “depression,” and three types of factors of the medical model are borrowed to illustrate as follows: first, biological factors, such as “fertility,” physiological health, and hormone fluctuations; second, psychological factors, including emotional processes and individual psychological characteristics of different genders; and finally, social factors, such as different social roles of male and female, employment environment, and so on.

### Biological causes analysis of corpus data

Some biological basis will also affect the occurrence and development of depression for the genetic basis. As can be observed from Figure 2, collocation scores of “adolescence,” “teen,” and “teenager” with the meaning of “youth” are at high levels, 5.45, 5.15, and 4.58, respectively, indicating that depression would occur early in adolescence. Furthermore, gender differences in depression first appeared in adolescence (Solomon and Herman, 2009). From the perspective of biological factors, the main reason for the difference is the different hormone changes of different genders at the beginning of adolescence period. Previous studies have suggested that hormone changes can affect people’s ability to cope with stress, resulting in dysfunction and prone to depression. From early youth to menopause, women’s hormone changes are more frequent than men’s. On the other hand, women’s unique physiological period from puberty brings them a certain degree of physiological and life burden. These burdens further stimulate and aggravate women’s emotional ups and downs, making women more likely to be negative and depressive than men.

As shown in Figure 3, “postpartum” and “postnatal” are ranked in the second and fifth highest places with collocation scores of 9.72 and 8.13. Both terms are related to “childbirth” (as well as the following word “post-natal” and “post-partum”), reflecting the unique biological characteristics of women: fertility and production. Similar words like “perinatal” and “natal” related to “childbirth” also rank prominently in Figure 3. The word “maternal” means “characteristic of a mother, implying a semantic relationship with “childbirth.” The words in the list that are closely related to women’s childbirth and production mainly reveal that the biological feature of “childbirth and production” is one of the inducements for women to have a higher probability of depression than men. From the perspective of external dominant factors, compared with men, most women will undergo the process of childbirth. From the view of internal hidden factors, women’s physical state would be more fragile during pregnancy and endocrine changes would be more drastic. These factors would bring about threats to their mental health and emotional fluctuations (Xi and Zhang, 2011) and then may seriously develop into depression.

Besides, the collocation score of “Alzheimer” in Figure 2, only second to the previous social identity words, is closely related to “depression.” Relevant studies show that patients with Alzheimer’s may also suffer from depression, and the proportion of patients with Alzheimer’s and depression simultaneously is about 30–50% (Frieden and Garai, 2012); among patients with Alzheimer, the number of women is also more than that of men, and the proportion is also close to 1:2. Moreover, the word “insomnia” appearing in Figures 3, 4 also troubles more women than men according to medical research (Frieden and Garai, 2012). In short, the incidence rate of Alzheimer, insomnia, and other diseases related to depression between men and women would indirectly result in a higher possibility of depression in women. Besides, the collocation of “bipolar” and “depression” in Figure 3 refers to bipolar depression, also known as bipolar disorder. Compared to monopolar depression shown by “unipolar” in the figure, bipolar depression not only has depressive episodes but also is accompanied by manic symptoms. The prevalence of Bipolar Disorder I is almost equal between men and women, while rapid-circulated Bipolar Disorder II is more common in women. However, the prominent symptom of most male patients of Bipolar Disorder II is mania, while most female patients are depressive, due to which some women with bipolar disorder are prone to develop depression, and the occurrence of bipolar disorder can also be one of the factors that make women more depressed than men.

Through comparative analysis, biological factors, such as fluctuation in hormone levels, fertility, and production, and diseases related to depression are found to increase the possibility of women suffering from depression no matter directly or indirectly.

### Psychological causes analysis of corpus data

Psychological factors are essential as depression is a psychological disease. In Figures 3, 4, the word “anxiety” ranks top, with a lexical frequency of 19,587 and 22,605, respectively. By further searching of the corpus, “anxiety” is a juxtaposition of “depression,” indicating that anxiety and depression often happen simultaneously. Previous researchers have only described the differences in prominent symptoms between male and female patients of depression, pointing out that female patients mainly showed irritability, systemic symptoms, somatic anxiety, and sleep disorders (Liang et al., 2013). In the co-occurrence context of “anxiety” and “depression” presented by the enTenTen20 corpus, the frequency of “he” was 376 while “she” was 492, revealing that anxiety often bothers more women than men. In addition, compared with men, women are more concerned about others’ evaluation of themselves and need more affirmation and recognition from others. Particularly, the tolerance of women for some negative evaluations is weaker than that of men (Lan and Zhang, 2015). The more sensitive psychological and instinctive characteristics of women also make women more subject to anxiety which then results in more serious depression. The above corpus evidence, combined with relevant medical research results, corroborates that women’s anxiety is more likely to trigger depression.

Second, in Figure 4, the collocation score of “anger” is 7.29, ranking fourth, namely, “depression” is often used to modify “anger.” Based on the above analysis of biological factors such as hormone level changes, fertility, and production, the female emotional instability tendency is significantly greater than that of the male. And the emotional fluctuations that change quickly will bring more threats to the mentality, with anger, tension, and even other more intense and complex emotional representations. Under this mechanism, the term “anger” is more closely related to women. Hence, this corpus provides evidence that the anger of women could help to induce depression.

Furthermore, according to Figures 3, 4, the collocation score and frequency of “PTSD (Post-Traumatic Stress Disorder)” are remarkable, which are 856 and 616, and 6.01 and 6.85, respectively, whether modified by “depression” or modifiers of “depression.” PTSD refers to the stress-related disorder that occurs later when a person is faced with strong mental stimulation. Previous studies have shown that the anti-stress capability of women is weaker than that of men (Su et al., 2008; Zhang et al., 2021). There is a significant gender difference in the incidence rate of PTSD after traumatic stress and women are two times as likely as men (An and Chen, 2014). And the frequency of the word “disorder” in Figures 3, 4 is as high as 4,114 and 5,956, respectively, indicating that the disorder is closely related to the occurrence of depression. From the view of psychological characteristics, women’s psychological state is relatively delicate, and psychological conflicts happen more often. In the response to various stimuli, women torture themselves with more stress disorders than men. According to the above data, stress or disorders in women further lead to a relatively high incidence of depression in women, that is, the more delicate and sensitive psychological characteristics of women than men become one of the catalysts of depression.

The above analysis could be concluded that female emotional states of anxiety, anger, disorder, and more sensitive psychological characteristics would evolve into triggers for depression in women.

### Social causes analysis of corpus data

Human beings all live in a society, where people are all constantly shaped by social construction, a long-term process in which understandings of such social factors as gender, race, class, and disability, are socially constructed. Once constructed, these factors would be viewed as natural or normal in society. Both individuals and groups develop themselves under the influence of social construction imperceptibly and unconsciously. For example, the notion of gender is molded by the saying “one is not born a woman, but becomes one” from Beauvoir. Currently, both men and women are still playing their roles in such normal forms, so depression caused by social factors such as the understanding of gender cannot be ignored. In Figure 2, the collocation score of “caregiver” is the highest, with a score of 5.75. Under the traditional notion of “males are breadwinners and females care for the family” constructed in Chinese society, the role of “caregiver” is supposed to play by women. The collocation scores and frequency of other words such as “mother,” “mum,” “mom,” and “wife” are 4.63, 4.61, 4.33, 3.61, and 332, 10, 34, 67, describing the role of women in family or society. In contrast, words collocated with “depression” as “Dad,” “husband,” and “father” corresponding to the role of men in family or society, with a lower score and frequency of 3.59, 3.18, 2.83, and 21, 54, 119, could also be found in Figure 2. The above comparative analysis shows that the main roles of women in society or family lead them to face a higher possible threat of depression.

Furthermore, referring to Figure 2, some other words such as “adolescence,” “teen,” “spouse,” “partner,” and “parent” referring to either male or female are all related to various social and family roles, following the above social roles as causes of depression. From a social perspective, different roles mean different responsibilities; and responsibilities often go with pressure. Moreover, by analyzing the repeated word “stress” collocated with “depression” in Figures 3, 4, the total lexical frequency of this word is as high as 6,257, suggesting that stress is closely related to depression. From the above-mentioned words with high frequency and high collocation score in Figures 2–4, we know that both men and women are under the pressure of these roles to varying degrees, resulting in more serious emotional problems. Nevertheless, through comparison of specific data, the roles of women are found to make them more subject to depression than men. By analyzing the social identity construction of men and women, the roles of women such as “mother” and “wife” are defined with more connotations and responsibilities. Traditional society stereotypes women with a relatively fixed image of roles; women’s identity choices are narrowed and restricted, and they bear more complex pressures to ease. Under the current employment environment, the employment opportunities and income of women are generally lower than that of men, suggesting that women are not only assuming family pressure but also undergoing much working pressure. The intensity of such dual pressure is crucial in inducing depression. Accordingly, such social factors as social and family roles and employment environment are also important factors leading to female depression.

Theoretically speaking, the above discussion and analysis may suggest that the study of depression with the help of a linguistic corpus would be inspiring for researchers to seek more supportive references. Practically, female depression could be probably intervened and prevented earlier if women and the people around them are much more aware of alleviating the possible causes.

## Conclusion

To sum up, the reasons for the differences in depression between women and men lie in that women are more liable to suffer from emotional problems and further develop depression than men under the influence of biological basis, psychological characteristics, and some social environment.

Based on the enTenTen20 network corpus, the up-to-date, authentic, and reliable data, the analysis and the conclusion are credible and of great social significance. An in-depth discussion of the causes of gender differences in depression may bring some inspiration for the prevention and alleviation of depression to a certain extent. The above analysis made is to put forward the following suggestions: first, the causes explored including unique physical burden and emotional states would help the public understand and care for more female depression sufferers; second, as for the causes of family roles, employment environment may be balanced and improved.

Besides the medical data, there must be some useful data from other fields worth studying, and this study may serve as a complement to solve the problem of depression due to the analysis of the supportive data in the linguistic corpus. As relevant data are analyzed from the sociological perspective, hopefully, the findings may offer some references or suggestions to the medical empirical research of depression soon.

## Data availability statement

The raw data supporting the conclusions of this article will be made available by the authors, without undue reservation.

## Author contributions

TZ contributed to the review concept and design. Both authors contributed to the article and approved the submitted version.
